# Supplementary material for: Selective Inhibition of PDE4B Reduces Binge Drinking in Two C57BL/6 Substrains
Source: Int J Mol Sci. 2021 May 21;22(11):5443. doi: 10.3390/ijms22115443 (PMC8196757; doi:10.3390/ijms22115443)
Supplement: Supplementary file 1 [file ijms-22-05443-s001.zip › ijms-1212378-supplementary.pdf]

Supplemental Results

Experiment 1: Assessment of A33 effects on alcohol-induced changes in motor activity in alcohol/A33-experienced mice.

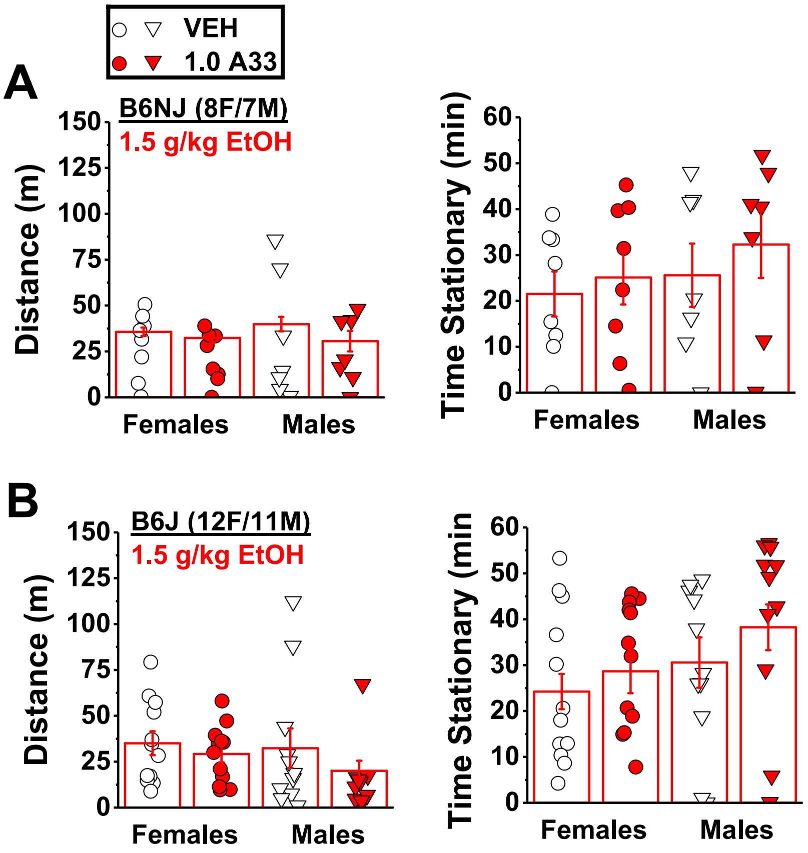

Suppl. Figure S1. Effect of A33 on alcohol-induced locomotor activity. Summary of the effect of vehicle (VEH) or A33 pretreatment (30 min earlier) upon **(A)** the total distance traveled and time spent stationary by B6NJ mice during a 2-h test post-injection with 1.5 g/kg of alcohol (EtOH). **(B)** Same as Panel A for B6J mice. The data represent the mean ± SEM of the number of mice indicated, in addition to the individual datapoints for each experimental group. *p<0.05 vs. 0 mg/kg of A33 (main effect); +p<0.05 vs. females (main effect).
